# Internal rotational patellar resection and patella alta induced patellar maltracking in total knee arthroplasty: intraoperative measurement of the patellofemoral pressure

**DOI:** 10.1186/s43019-024-00231-8

**Published:** 2024-08-22

**Authors:** Sanshiro Yasuma, Sakurako Kato, Takuya Usami, Yusuke Hattori, Yuji Joyo, Hiroo Shiraga, Masahiro Nozaki, Hideki Murakami, Yuko Waguri-Nagaya

**Affiliations:** 1 Department of Orthopaedic Surgery, Nagoya City University East Medical Center, 2-23 Wakamizu 1, Chikusa, Nagoya, Japan; 2 Department of Orthopaedic Surgery, Nagoya City University, Kawasumi 1, Mizuho-Cho, Mizuho, Nagoya, Japan

**Keywords:** Lateral retinacular release, Patellar height, Patellar resection angle, Patellar tracking, Patellofemoral pressure, Total knee arthroplasty

## Abstract

**Background:**

Anterior knee pain due to patellar maltracking following total knee arthroplasty (TKA) reduces patients’ satisfaction. This study aimed to determine the patellofemoral pressure (PFP) in patients with favorable patellar tracking (FT) and impaired patellar tracking (IT) following TKA, the factors causing patellar maltracking, and the effect of lateral retinacular release (LRR) on patients with IT.

**Methods:**

Forty-four patients with varus knee osteoarthritis undergoing cruciate-retaining TKA were enrolled. After component implantation, patients with a separation of ≥ 2 mm of the patellar medial facet from the medial femoral trochlea throughout knee range of motion were classified into the IT group; meanwhile, the others were classified into the FT group. PFP was measured intraoperatively in three phases: (1) with the resurfaced patella (RP); (2) with the resurfaced patella and knee (RPK); and (3) when LRR was performed in IT (post-LRR). The PFPs at 0°, 90°, 120°, and 135° knee flexion were compared between FT and IT using the Mann–Whitney *U* test. Pairwise comparison of the PFP in IT between RPK and post-LRR was performed using the Wilcoxon signed-rank test. Correlations between PFP and pre- and postoperative radiographic parameters, such as hip–knee–ankle angle, lateral distal femoral angle, medial proximal tibial angle, anterior femoral offset, Insall–Salvati ratio (ISR), patellar tilt, and patellar resection angle (PRA), were evaluated using Spearman’s rank correlation coefficients.

**Results:**

High lateral PFP in the knee flexion position led to patellar maltracking. Patients with IT (*n* = 24) had higher lateral and lower medial PFP than did patients with FT (*n* = 20) at 90°, 120°, and 135° knee flexion in RP and RPK. LRR in IT reduced the lateral PFP in the knee flexion position. PRA and ISR were correlated with the lateral PFP at no less than 90° in RP and RPK.

**Conclusions:**

This study demonstrated that internal rotational patellar resection, which resulted in a thick medial patellar remnant and a thin lateral counterpart, and patella alta were the causative factors of high lateral PFP, which induced patellar maltracking after TKA. Surgeons should avoid internal rotational patellar resection to achieve FT and perform LRR in patients with patellar maltracking.

## Background

Total knee arthroplasty (TKA) is regarded as the most successful procedure for reducing pain and improving the quality of life in patients with knee osteoarthritis (OA). However, up to 19% of patients who undergo primary TKA are not satisfied [1]. Anterior knee pain (AKP) is one of the most common complications that reduce patient satisfaction after TKA. Although AKP has multifactorial causes [2], patellar maltracking is a known cause that accounts for 8–27% of the incidence of AKP after TKA [3, 4]. Patellar maltracking is caused by various factors, including prosthetic design, surgical approach, component alignment, and patellar height [5]; of these, component alignment is a major factor that affects patellar tracking in TKA. Coronal, axial, and sagittal malalignments of components cause patellar maltracking [5–8]. A previous biomechanical study demonstrated that increased lateral patellofemoral pressure (PFP) caused by excessive tension in the lateral retinacular tissue induces patellar maltracking [9, 10]. Furthermore, patellar alta is associated with high PFP during knee flexion and, thus, is a causative factor of patellar maltracking after TKA [11, 12].

To achieve optimal patellar tracking in TKA, it is important not only to achieve correct alignment [5–8] but also to perform lateral retinacular release (LRR) in the case of patellar maltracking after the implantation of components [13, 14]. Previous cadaveric studies have demonstrated that LRR improves patellar maltracking by reducing lateral retinacular tension and consequently achieving a physiological balance between the lateral and medial PFP [9, 10]. However, the intraoperative assessment of patellar tracking in a clinical situation is generally conducted on the basis of manual examinations, such as the “no-thumb test” and “towel clip test” [15, 16]. Therefore, whether additional LRR should be performed after the implantation of TKA component and the magnitude of the effect of LRR on patellar tracking depend on the surgeon’s subjective evaluation. No previous study has performed a clinical quantitative assessment of patellar tracking in TKA by measuring the balance of the PFP and investigating the effect of LRR on PFP. Such a quantitative evaluation is preferable for developing a more accurate and consistent assessment of patellar tracking. Therefore, the present study aimed to (1) quantitatively evaluate the effect of LRR on patellar tracking in TKA and (2) elucidate the characteristics of component alignment and anatomy that cause patellar maltracking in a clinical situation. We hypothesized that patients exhibiting patellar maltracking after the implantation of TKA components have high lateral PFP and that subsequent LRR improves patellar maltracking by reducing the lateral PFP.

## Methods

### Participation

This retrospective case–control study was conducted in accordance with the principles embodied in the World Medical Association’s 1964 Declaration of Helsinki and its 2013 revision [17] and was approved by the Institutional Review Board and Ethics Committee of Nagoya City University with registration number 21-03-338. All patients provided oral and written informed consent for participation. The inclusion criteria were pain and functional disorder due to substantial varus knee OA, classified as Kellgren–Lawrence (KL) grade 3 or 4 [18]. The exclusion criteria were mild varus knee OA (KL grade 1 or 2), rheumatoid arthritis, valgus knee OA, and revision TKAs.

**Fig. 1:**
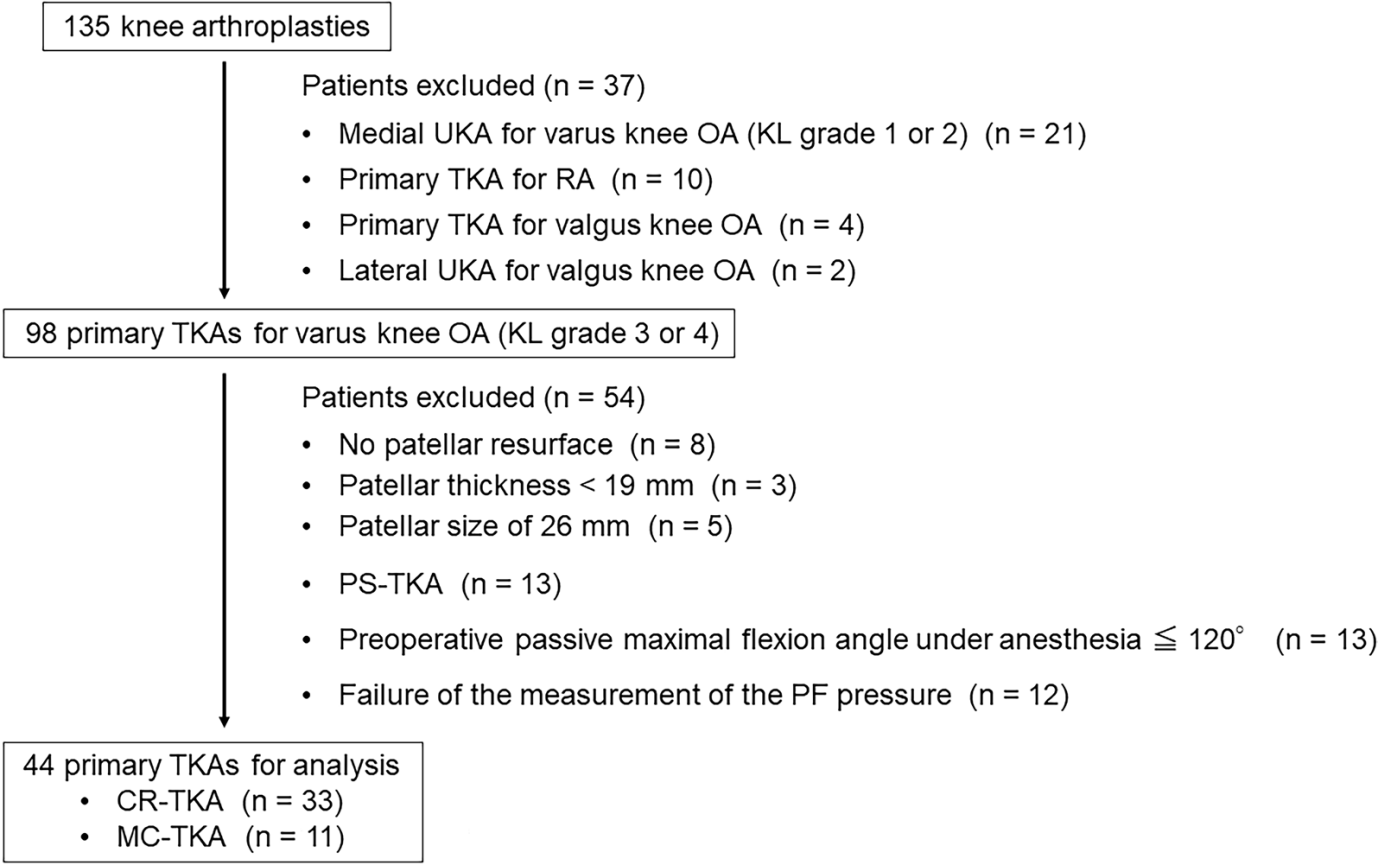
Flowchart of patient selection. *CR* cruciate-retaining, *KL* Kellgren–Lawrence, *MC* medial-congruent, *OA* osteoarthritis, *PF* patellofemoral, *PS* posterior-stabilized, *RA* rheumatoid arthritis, *TKA* total knee arthroplasty, *UKA* unicompartmental knee arthroplasty

A total of 135 consecutive knee arthroplasties were performed at our hospital between September 2019 and August 2022. Overall, 37 patients were excluded, and 98 primary TKAs for substantial varus knee OA were consequently included in this study (Fig. 1). All TKAs were performed using the Persona Knee System^®^ (Zimmer Biomet, Warsaw, IN, USA) with cement. Furthermore, the exclusion criteria based on the intraoperative findings were as follows: (1) patellar resurface was not performed because the patellar cartilage damage was identified as mild (International Cartilage Repair Society grade 2 or lower) [19]; (2) the patellar thickness measured using a caliper was < 19 mm; (3) the implanted patellar component measured 26 mm in size; (4) posterior-stabilized (PS)-type prostheses were implanted; (5) the preoperative passive maximal flexion angle of the knee under general anesthesia was no more than 120°; (6) intraoperative measurement of the PFP could not be properly performed because of the poor fixing property of the custom-made patellar component to the bone resection surface of the patellar remnant. After applying these exclusion criteria, 44 cases comprising 33 cruciate-retaining (CR)-type bearing and 11 medial-congruent (MC)-type bearing TKAs were included in the analysis (Fig. 1).

### Surgical procedures

All 44 TKAs were performed under the direct supervision of the senior author (Y.W.N.), using a tourniquet under general anesthesia. In all cases, a medial parapatellar arthrotomy was used for exposure, and both the femur and tibia were resected perpendicular to the mechanical axis in the coronal plane using portable navigation “KneeAlign^®^ 2” (Orthalign, Aliso Viejo, CA, USA). The distal femoral resection was set at 3° flexion to the femoral mechanical axis, with the same thickness as that of the distal femoral component. Subsequently, a tibial osteotomy was performed with a thickness of 10 mm from the center of the articular surface of the lateral plateau with a 7° posterior slope, attempting posterior cruciate ligament (PCL) retention with the bone island. Femoral component rotation was decided on the basis of the posterior condylar angle (PCA), namely the angle of the surgical epicondylar axis with respect to the posterior condylar line on preoperative axial computed tomography (CT) image [20]; the PCA was 3.3 ± 1.1° of external rotation, ranging from 1.2° to 4.9° in our cases. A previous systematic review stated that the femoral component rotation based on the posterior condylar line [= femoral component rotational angle (FCRA)] should be 2° ≤ external rotation ≤ 5° to obtain preferable clinical outcomes [21]. Therefore, we set the planned FCRA as 3° or 4° of external rotation considering a potential difference of ± 1° between the planned FCRA and the obtained value using a conventional femoral cutting guide [22]. We set a FCRA of 3° external rotation for 33 cases in which PCA was less than 4°, and a FCRA of 4° external rotation for the remaining 11 cases. The femoral component position was determined using the anterior referencing system. The level of femoral condyle osteotomy in the coronal plane was set at the proximal anterolateral margin to avoid anterior femoral notching. According to the bone surface gap at 0° and 90° knee flexion with applied tension force of 100 N to the center of the femorotibial joint using a Versatile Tensor (Zimmer Biomet, Warsaw, IN, USA), the size of the femoral component was determined to compensate the difference in the gap between 0° and 90° flexion, as previously described [23]. The femoral trial component was then inserted, followed by measurement of the component gap in the same manner as the bone surface gap. MC-type bearing was chosen instead of the CR-type bearing when mid-flexion instability was demonstrated, and the component gaps at 30° and 60° knee flexion were greater than those at 0° flexion by no less than 3 mm [24]. The thickness of bearing was determined to match the component gap at 0° flexion. The tibial component was aligned along the line connecting the center of the PCL insertion to the medial third of the tibial tuberosity. Osteophytes were removed as much as possible, and the minimum necessary release of the deep medial collateral ligament was performed to balance the lateral and medial gaps.

### PFP measurement

The PFP was measured using a custom-made dome-shaped patellar component, which had the same shape as the original patellar component (32 mm in diameter) and incorporated two ultrathin (200 µm force sensors (FlexiForce^®^, Nitta Corp., Osaka, Japan). Three spikes on the back of the patellar component enabled fixation to the bone resection surface of the patellar remnant. One force sensor was embedded on the medial side of the patellar component, and the other in its lateral counterpart, which made it possible to measure the pressure on the patellar component separated into the medial and lateral compartments. The force sensor could measure pressures of up to 110 N. The PFP, which was captured by the force sensor at 8 Hz intraoperatively, was transduced to real-time data displayed on a laptop computer using a software (Flexiforce^®^ ELF system version 4, Nitta Corp., Osaka, Japan).

### Intraoperative measurement

**Fig. 2:**
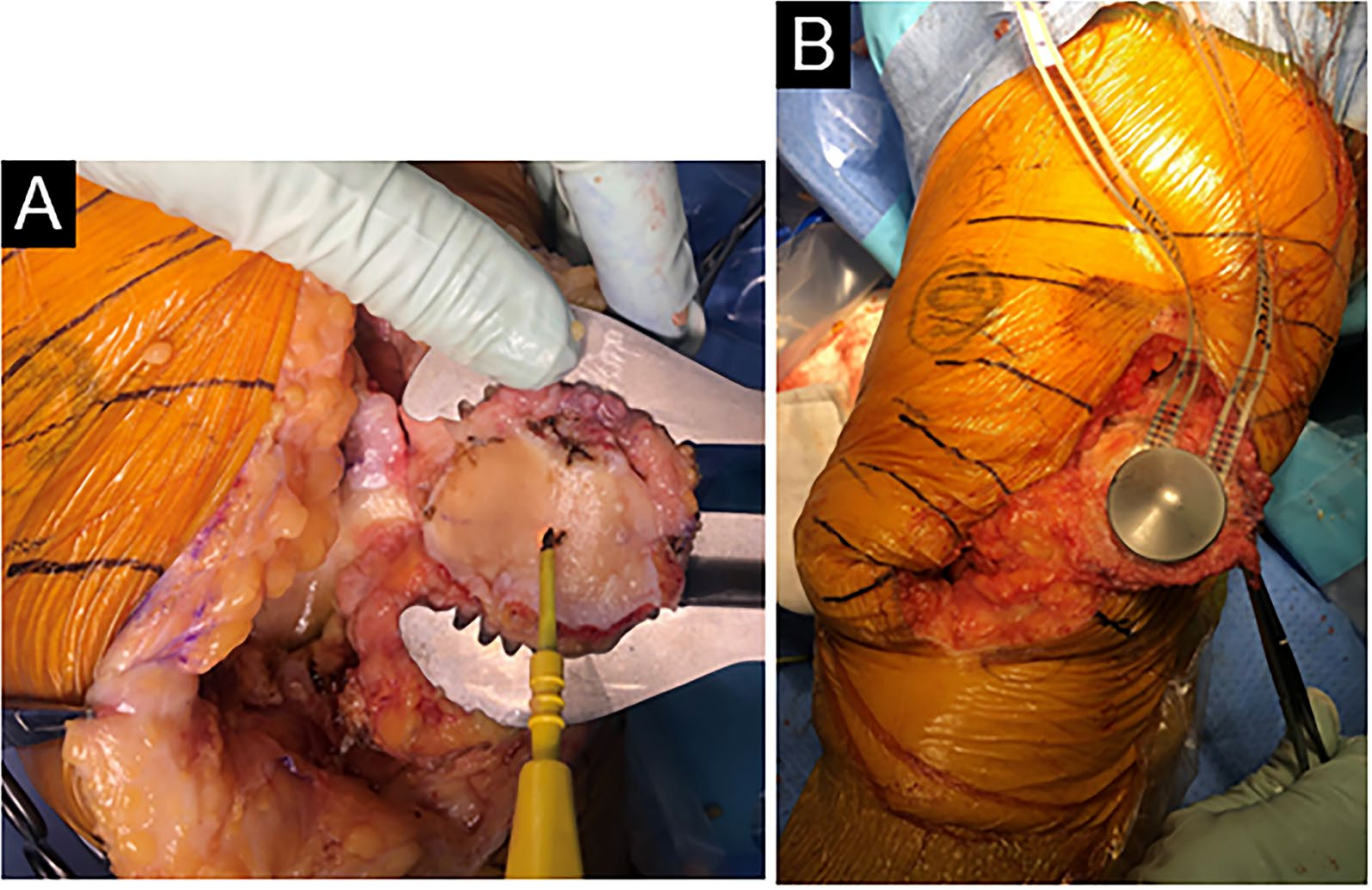
Intraoperative measurement of the PFP in the left knee. Prior to patellar osteotomy, we marked the center, the most proximal and most distal points of the patellar median ridge, and the points where the mediolateral axis of the patella intersected both ends of the patella. The marked points were used as landmarks after patellar osteotomy (**A**). We adjusted the center of the custom-made patellar component to the center of the resected patellar median ridge; additionally, we aligned the medial and lateral pressure sensors embedded in the patellar component with the mediolateral axis of the patella (**B**). *PFP* patellofemoral pressure

**Fig. 3:**
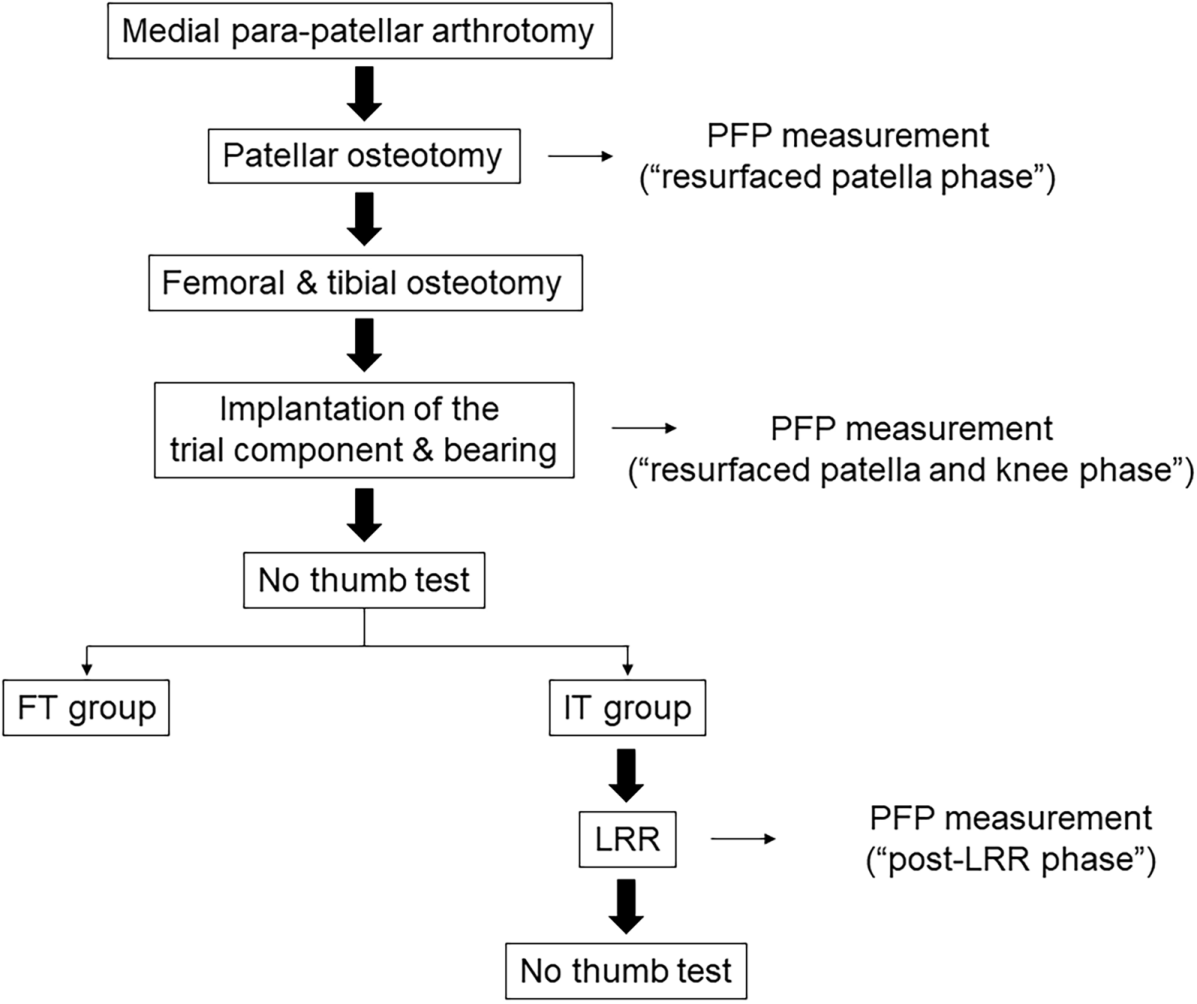
Schematic of the experimental protocol. Cases with < 2-mm separation of the medial patellar facet from the medial femoral trochlea during the “no-thumb test” were classified into the FT group, whereas those with no less than 2-mm separation were classified into the IT group. *IT* impaired patellar tracking, *FT* favorable patellar tracking, *LRR* lateral retinacular release, *PFP* patellofemoral pressure

First, patellar osteotomy was performed using a zig after a medial parapatellar arthrotomy. The amount of patellar osteotomy measured using a caliper was set to the same thickness as the original patellar component, and caution was taken to ensure that the thickness of the patellar remnant after resection was no less than 12 mm. Subsequently, we fixed the custom-made dome-shaped patellar component to the bone resection surface of the patellar remnant at the center of the patellar median ridge and aligned the medial and lateral pressure sensors embedded in the component with the mediolateral axis of the patella (Fig. 2A, B). The joint capsule was temporarily sutured at three places in the extended-knee position, followed by the first measurement of the PFP, which was defined as the “resurfaced patella (RP) phase.” The lateral and medial PFPs were separately measured at 0°, 90°, 120°, and 135° knee flexion in a steady state. When the flexion contracture existed, we measured the PFP in the maximal extended-knee position instead of 0° knee extension, whereas we measured the PFP in the maximal flexed-knee position instead of 135° flexion when the knee could not be flexed to these degrees. The second measurement of the PFP was performed in the same manner as for the first one after the implantation of the trial components and bearing, which was defined as the “resurfaced patella and knee (RPK) phase.” Subsequently, three temporal sutures at the capsule were cut, and the “no-thumb test” was conducted to evaluate patellar tracking based on a previous study [14, 16]. Briefly, we classified all patients into the favorable patellar tracking (FT) group or the impaired patellar tracking (IT) group according to the amount of separation of the medial patellar facet from the medial femoral trochlea during full extension to possible flexion of the knee. Patients with a separation of less than 2 mm were classified into the FT group, whereas those with a separation of ≥ 2 mm were classified into the IT group. All cases classified into the IT group underwent subsequent LRR, followed by the third measurement of the PFP, which was defined as the “post-LRR phase,” and by a revaluation of patellar tracking with the “no-thumb test.” LRR was performed using an inside-out technique in which the lateral retinacular tissues from 20 mm proximal to the superior pole of the patella to the level of the middle of the patella were resected. Specifically, the joint capsule and deep transverse fibers of the iliotibial band, termed iliotibial band-patellar fibers, were resected using electric cautery along the lateral border of the vastus lateralis obliquus proximally and 10 mm lateral to the lateral margin of the patella distally [25]. A schematic of the experimental protocol is shown in Fig. 3.

### Radiographic measurements

**Fig. 4:**
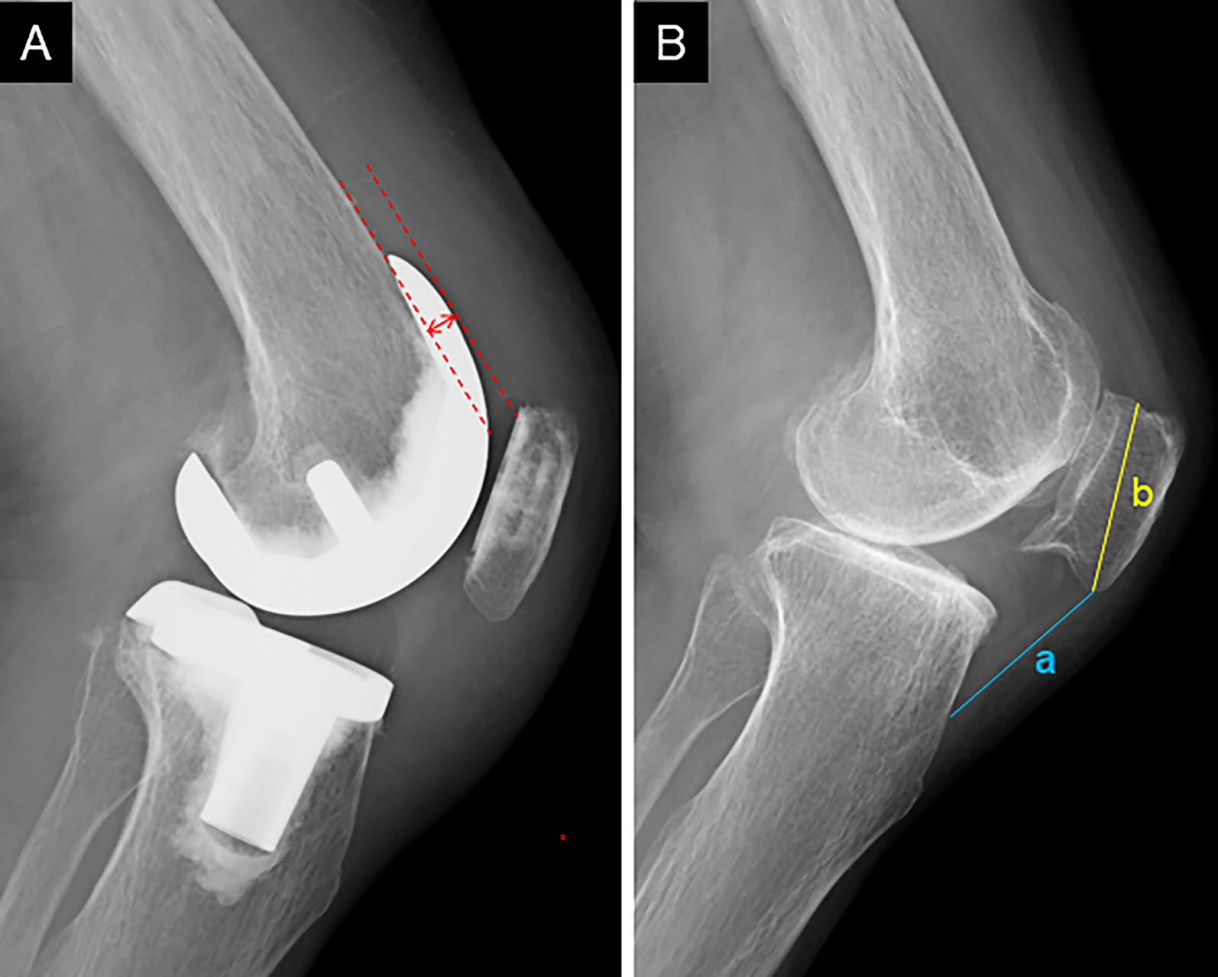
Radiographic measurement using a lateral knee radiograph. **A** AFO was defined as the distance between the anterior femoral cortex and the anterior aspect of the femoral condyle or femoral component (red double-headed arrow). **B** ISR was defined as the ratio of the distance between the distal pole of the patella and the tibial tuberosity (a) to the distance between the distal and proximal poles of the patella (b). *AFO* anterior femoral offset, *ISR* Insall–Salvati ratio

**Fig. 5:**
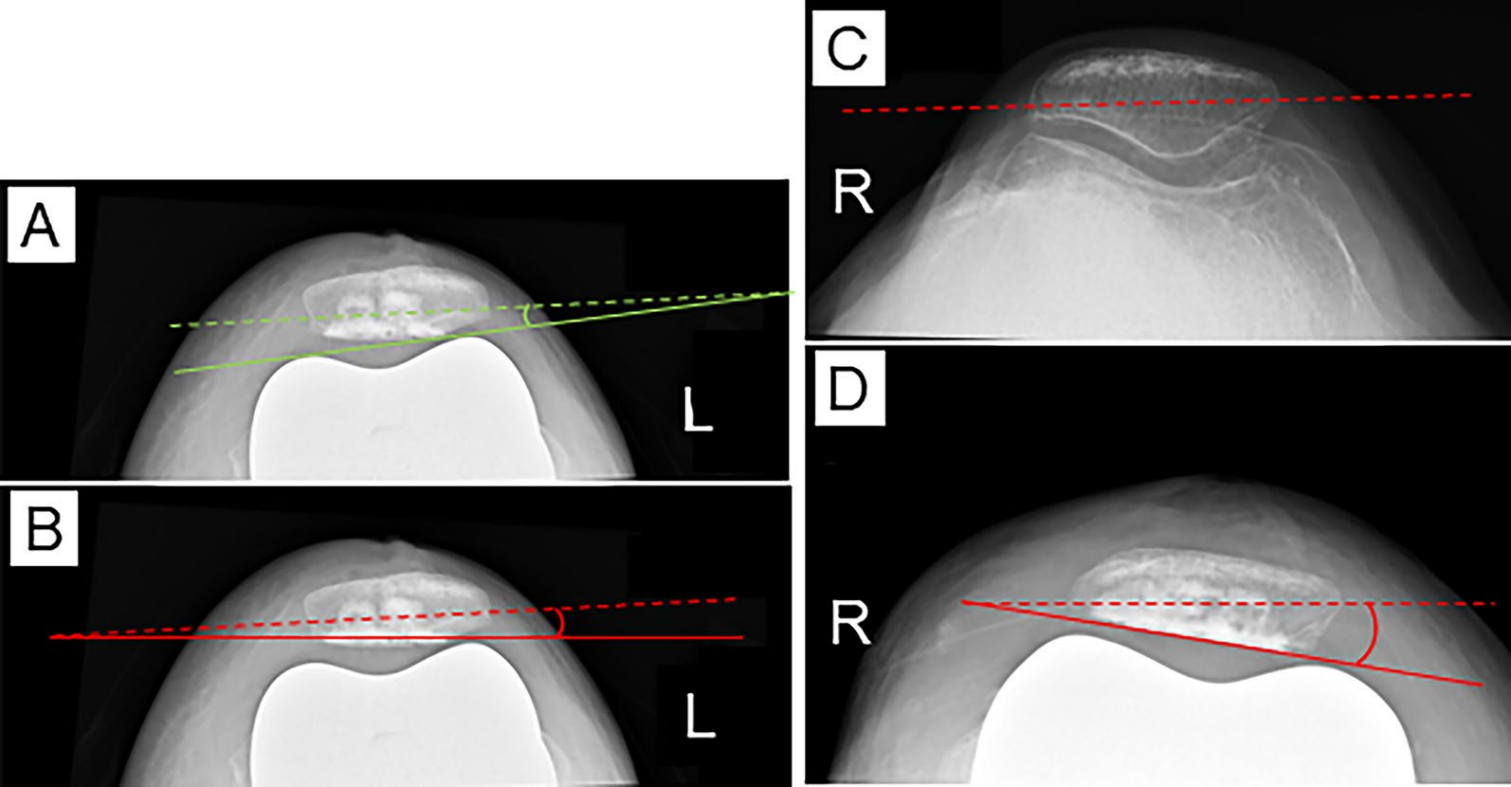
Radiographic measurement using the skyline view. **A** The PT is defined as the angle of a line drawn along the long axis of the patella (green dashed line) with respect to a line connecting the anterior aspect of the femoral condyles or femoral component (green solid line), with an external rotational angle being considered as positive. Postoperative PT of the left knee is shown in **A**. **B**–**D** The PRA was defined as the angle of a line drawn through the patellar prosthesis–bone interface (red solid line) with respect to a line along the long axis of the patella (red dashed line), with an external rotational angle being considered as positive. The PRA of the left knee was 2.3° (**B**). Based on the pre- and postoperative radiographs of the right knee, the PRA was calculated as −8.9° (**C**, **D**). *PRA* patellar resection angle, *PT* patellar tilt

All patients underwent pre- and postoperative (at 2 weeks after surgery) radiographs. Radiographic measurement was performed for a battery of radiographs, including a full-leg-length standing anteroposterior radiograph, a lateral knee radiograph at 45° flexion, and a skyline view at 60° flexion. The hip–knee–ankle angle, lateral distal femoral angle, and medial proximal tibial angle were measured on a full-length standing anteroposterior radiograph [26]. Anterior femoral offset (AFO) and the Insall–Salvati ratio (ISR) [27] were measured on a lateral knee radiograph (Fig. 4). AFO was defined as the distance between the anterior femoral cortex and anterior aspect of the femoral condyle [28]. On a skyline view, patellar tilt (PT) and patellar resection angle (PRA) were measured (Fig. 5). PT was defined as the angle between the line connecting the anterior aspect of the femoral condyles and the line along the long axis of the patella [29]. The long axis of the patella was determined on preoperative radiographs using the medial-divot method, as previously described [30]. The PRA was defined as the angle between a line along the long axis of the patella and a line drawn through the patellar prosthesis–bone interface [31]. Two orthopedic surgeons with considerable experience in knee arthroplasty surgery retrospectively performed the radiographic measurements and independently evaluated the radiographs of patients twice with a 6-week interval between the first and second evaluations to assess intra- and interrater reliabilities. The radiographs for measurement were randomized, and both testers were blinded to the patients’ operative findings.

Additionally, we investigated the postoperative change in the femoral rotational axis from the preoperative value operation by calculating the difference between the planned FCRA and PCA (FCRA − PCA).

### Statistical analysis

The medial and lateral PFPs captured by the force sensors for 3 s in a steady state were averaged separately and analyzed using SPSS Statistics version 28.0.1 (IBM, Corp., Armonk, NY, USA). The PFP in the RP and RPK phases was compared between the FT and IT groups using the Mann–Whitney *U* test. A pairwise comparison of the PFP in the IT group between the RPK and post-LRR phases was performed using the Wilcoxon signed-rank test. Additionally, correlations between the PFP and the radiographic measurement parameters were evaluated for the RP and RPK phases by calculating Spearman’s rank correlation coefficients (*rs*). Statistical significance was set at *p* < 0.05. Intra- and interrater reliabilities of each radiographic parameter were assessed by calculating the intraclass correlation coefficient (ICC). A post-hoc power analysis was performed using G*Power version 3.1.9.7 (Heinrich Heine University, Dusseldorf, Germany), which indicated that our sample size of 44 cases achieved a power of 82%, with a two-sided alpha of 0.05.

## Results

Among the 44 enrolled patients, 20 patients were classified into the FT group and 24 into the IT group. Demographic characteristics did not show any significant difference between the two groups (Table 1). The difference between the thickness of the native patella and the combined thickness of the patellar remnant and patellar component during PFP measurement [thickness of the patellar remnant after osteotomy + patellar component (8.5 mm) − native patella] was −0.2 ± 1.7 mm for the FT group and 0.2 ± 1.0 mm for IT group.

**Table 1:** Demographics of the patients classified into groups

Patients	FT group (*n* = 20)	IT group (*n* = 24)	*p* value
Age (years)	75.1 ± 5.5	75.8 ± 5.9	0.80
Sex (female/male)	16/4	17/7	0.73
Height (cm)	154.5 ± 8.0	154.9 ± 7.8	0.83
Weight (kg)	62.2 ± 11.0	60.5 ± 13.0	0.65
BMI (kg/m^2^)	26.1 ± 4.6	25.0 ± 3.8	0.40
Knee ROM (°) (in the awake state)			
Flexion	117.0 ± 14.6	116.7 ± 15.2	0.95
Extension	−12.0 ± 5.7	−10.0 ± 8.4	0.11
KL grade (grade 3/grade 4)	6/14	11/13	0.36
Bearing type (CR/MC)	17/3	16/8	0.29

Continuous data are presented as mean ± standard deviation. The Mann–Whitney *U* test was used to compare the continuous variables between the two groups, whereas Fisher’s exact test was applied to compare the categorical variables

*BMI* body mass index, *CR* cruciate-retaining, *FT* favorable patellar tracking, *IT* impaired patellar tracking, *KL* Kellgren-Lawrence, *MC* medial-congruent, *ROM* range of motion

### Comparison of the PFP between FT and IT groups

The PFP in the RP and RPK phases in the FT and IT groups are listed in Table 2. The mean lateral PFP in the RPK phase was significantly higher for the IT group than for the FT group at 90° (48.6 ± 23.0 N versus 22.0 ± 11.5 N, *p* < 0.001), 120° (66.2 ± 19.1 N versus 34.6 ± 21.5 N, *p* < 0.001) and 135° (76.3 ± 22.5 N versus 42.5 ± 27.1 N, *p* < 0.001). Furthermore, the mean medial PFP in the RPK phase was significantly lower for the IT group than for the FT group at 90° (5.3 ± 6.2 N versus 9.4 ± 8.1 N, *p* = 0.029), 120° (7.6 ± 10.4 N versus 14.8 ± 12.8 N, *p* = 0.019) and 135° (10.2 ± 14.9 N versus 20.4 ± 16.1 N, *p* = 0.023).

**Table 2:** PFP of the RP phase and the RPK phase for the FT and IT groups

		Lateral PFP			Medial PFP		
		FT group	IT group	*p* value	FT group	IT group	*p* value
PFP of the resurfaced patella phase							
Knee flexion angle	0°	5.4 ± 4.4	4.2 ± 4.4	n.s.	3.4 ± 3.1	2.9 ± 3.3	n.s.
	90°	21.3 ± 18.1	38.8 ± 18.0	< 0.001	8.2 ± 9.0	5.1 ± 7.2	n.s.
	120°	24.8 ± 21.5	42.5 ± 20.6	0.002	10.8 ± 8.3	6.9 ± 9.0	n.s.
	135°	31.0 ± 24.3	49.7 ± 30.6	0.034	18.4 ± 12.3	9.8 ± 9.1	0.013
PFP of the resurfaced patella and knee phase							
Knee flexion angle	0°	2.8 ± 1.8	2.5 ± 2.1	n.s.	3.5 ± 2.9	3.2 ± 4.0	n.s.
	90°	22.0 ± 11.5	48.6 ± 23.0	< 0.001	9.4 ± 8.1	5.3 ± 6.2	0.029
	120°	34.6 ± 21.5	66.2 ± 19.1	< 0.001	14.8 ± 12.8	7.6 ± 10.4	0.019
	135°	42.5 ± 27.1	76.3 ± 22.5	< 0.001	20.4 ± 16.1	10.2 ± 14.9	0.023

Data are presented as mean ± standard deviation

*FT* favorable patellar tracking, *IT* impaired patellar tracking, *PFP* patellofemoral pressure, *RP* resurfaced patella, *RPK* resurfaced patella and knee, *n.s.* not significant

### Effect of LRR on the PFP in IT group

The PFPs during the RPK and post-LRR phases in the IT group are listed in Table 3. The mean lateral PFP in the post-LRR phase was significantly lower than that in the RPK phase at 90° (33.3 ± 16.9 N versus 48.6 ± 23.0 N, *p* < 0.001), 120° (46.1 ± 21.2 N versus 66.2 ± 19.1 N, *p* < 0.001) and 135° (59.9 ± 25.3 N versus 76.3 ± 22.5 N, *p* < 0.001). Out of 24 patients who underwent LRR, 18 achieved favorable patellar tracking during the “no-thumb test.” Meanwhile, we performed the extended LRR for the remaining six cases to further improve patellar tracking. The retinacular tissues from the mid-level of the patella distally to the joint line along the lateral border of the patellar tendon were resected using electric cautery according to a previous study [13]. Consequently, all of them achieved favorable patellar tracking during the “no-thumb test.”

**Table 3:** PFP of the RPK phase and the post-LRR phase for the IT group

	Lateral PFP (N)			Medial PFP (N)		
	RPK phase	Post-LRR phase	*p* value	RPK phase	Post-LRR phase	*p* value
Knee flexion angle						
0°	2.5 ± 2.1	2.2 ± 2.1	n.s.	3.2 ± 4.0	1.9 ± 2.0	n.s.
90°	48.6 ± 23.0	33.3 ± 16.9	< 0.001	5.3 ± 6.2	4.0 ± 4.2	n.s.
120°	66.2 ± 19.1	46.1 ± 21.2	< 0.001	7.6 ± 10.4	6.6 ± 6.1	n.s.
135°	76.3 ± 22.5	59.9 ± 25.3	< 0.001	10.2 ± 14.9	9.9 ± 9.2	n.s.

Data are presented as mean ± standard deviation

*IT* impaired patellar tracking, *LRR* lateral retinacular release, *PFP* patellofemoral pressure, *RPK* resurfaced patella and knee, *n.s.* not significant

### Correlations between the PFP and radiographic parameters

The parameters measured on pre- and postoperative radiographs and the ICC of each radiographic measurement are presented in Table 4. Both intra- and interrater reliabilities were excellent for each radiographic measurement according to the ICCs. The correlations between the radiographic measurement parameters and the lateral PFP in the RP and RPK phases are presented in Tables 5 and 6, respectively. The ISR of preoperative radiographs was significantly correlated with the lateral PFP in the RP phase at the knee flexion of 90° (*rs* = 0.38, *p* = 0.012), 120° (*rs* = 0.43, *p* = 0.0053) and 135° (*rs* = 0.31, *p* = 0.045), whereas the PRA was negatively correlated with the lateral PFP in the RP phase at the knee flexion of 90° (*rs* = −0.37, *p* = 0.015), 120° (*rs* = −0.56, *p* < 0.001) and 135° (*rs* = −0.60, *p* < 0.001) (Table 5). The ISR of postoperative radiographs was significantly correlated with the lateral PFP in the RPK phase at 90° (*rs* = 0.34, *p* = 0.030) and 120° (*rs* = 0.37, *p* = 0.018), whereas the PRA was negatively correlated with the lateral PFP in the RPK phase at 120° (*rs* = −0.47, *p* = 0.0015) and 135° (*rs* = −0.40, *p* = 0.0078) of knee flexion (Table 6). In contrast, there was no correlation between the medial PFP and the radiographic parameters of both pre- and postoperative radiographs. Furthermore, there was no significant correlation between (FCRA − PCA) and the lateral PFP at 90° (*rs* = 0.001, *p* = 0.99), 120° (*rs* = −0.08, *p* = 0.62) and 135° (*rs* = 0.02, *p* = 0.90) of knee flexion; in addition, there was no significant difference in (FCRA − PCA) between the FT and IT groups (−0.05 ± 0.64° versus 0.07 ± 0.95°, *p* = 0.90).

**Table 4:** Parameters measured using pre- and postoperative radiographs and the ICC of the radiographic measurement

	Preoperative radiographs (mean ± SD)	Postoperative radiographs (mean ± SD)	ICC (95% CI)	
			Intrarater ICC	Interrater ICC
HKA (°)	−12.3 ± 5.5	−1.0 ± 3.4	0.98 (0.95–0.99)	0.82 (0.62–0.91)
LDFA (°)	89.8 ± 2.4	90.6 ± 2.3	0.94 (0.86–0.98)	0.94 (0.87–0.97)
MPTA (°)	83.1 ± 3.3	90.1 ± 1.9	0.89 (0.75–0.96)	0.94 (0.90–0.97)
AFO (mm)	9.6 ± 2.9	5.8 ± 1.3	0.98 (0.96–0.99)	0.92 (0.85–0.96)
ISR	1.01 ± 0.13	1.08 ± 0.13	0.82 (0.60–0.92)	0.84 (0.61–0.94)
PT (°)	3.0 ± 3.4	4.0 ± 3.5	0.92 (0.82–0.97)	0.81 (0.66–0.90)
PRA (°)	–	2.0 ± 4.1	0.94 (0.78–0.98)	0.89 (0.75–0.96)

*AFO* anterior femoral offset, *CI* confidence interval, *HKA* hip-knee-ankle angle, *ICC* intraclass correlation coefficient, *ISR* Insall–Salvati ratio, *LDFA* lateral distal femoral angle, *MPTA* medial proximal tibial angle, *PRA* patellar resection angle, *PT* patellar tilt, *SD* standard deviation

**Table 5:** Correlations between the radiographic parameters and the lateral PFP of the RP phase

	Lateral PFP of the resurfaced patella phase (N)							
	0° knee flexion		90° knee flexion		120° knee flexion		135° knee flexion	
	*rs*	*p* value	*rs*	*p* value	*rs*	*p *value	*rs*	*p *value
Parameters of preoperative radiographs								
HKA (°)	−0.20	n.s.	−0.02	n.s.	−0.15	n.s.	0.05	n.s.
LDFA (°)	0.28	n.s.	−0.05	n.s.	0.00	n.s.	0.01	n.s.
MPTA (°)	−0.22	n.s.	0.12	n.s.	0.03	n.s.	0.04	n.s.
AFO (mm)	−0.19	n.s.	0.21	n.s.	0.22	n.s.	0.05	n.s.
ISR	−0.15	n.s.	0.38	0.012*	0.43	0.0053*	0.31	0.045*
PT (°)	0.02	n.s.	0.13	n.s.	0.27	n.s.	0.27	n.s.
Parameter of postoperative radiographs								
PRA (°)	0.24	n.s.	−0.37	0.015*	−0.56	< 0.001*	−0.60	< 0.001*

*AFO* anterior femoral offset, *HKA* hip–knee–ankle angle, *ISR* Insall–Salvati ratio, *LDFA* lateral distal femoral angle, *MPTA* medial proximal tibial angle, *PFP* patellofemoral pressure, *PRA* patellar resection angle, *PT* patellar tilt, *RP* resurfaced patella, *n.s.* not significant, *rs* Spearman’s rank correlation coefficients

Significant *p* values at this threshold are marked with an asterisk

**Table 6:** Correlations between the radiographic parameters and the lateral PFP of the RPK phase

	Lateral PFP of the resurfaced patella and knee phase (N)^†^							
	0° knee flexion		90° knee flexion		120° knee flexion		135° knee flexion	
	*rs*	*p* value	*rs*	*p* value	*rs*	*p* value	*rs*	*p* value
Parameters of postoperative radiographs								
HKA (°)	−0.30	n.s.	0.01	n.s.	−0.02	n.s.	−0.02	n.s.
LDFA (°)	0.39	0.010*	−0.01	n.s.	0.05	n.s.	0.08	n.s.
MPTA (°)	−0.22	n.s.	0.24	n.s.	0.21	n.s.	0.24	n.s.
AFO (mm)	−0.04	n.s.	0.29	n.s.	0.16	n.s.	0.10	n.s.
ISR	−0.08	n.s.	0.34	0.030*	0.37	0.018*	0.30	0.053
PT^†^ (°)	0.14	n.s.	0.14	n.s.	0.14	n.s.	0.15	n.s.
PRA (°)	0.18	n.s.	−0.24	n.s.	−0.47	0.0015*	−0.40	0.0078*

*AFO* anterior femoral offset, *FT* favorable patellar tracking, *HKA* hip–knee–ankle angle, *ISR* Insall–Salvati ratio, *IT* impaired patellar tracking, *LDFA* lateral distal femoral angle, *LRR* lateral retinacular release, *MPTA* medial proximal tibial angle, *PFP* patellofemoral pressure, *PRA* patellar resection angle, *PT* patellar tilt, *RPK* resurfaced patella and knee, *n.s.* not significant, *rs* Spearman’s rank correlation coefficients

^a^With regard to PT, correlation was evaluated with the lateral PFP of the RPK phase for the FT group and the lateral PFP of post-LRR phase for the IT group

Significant *p* values at this threshold are marked with an asterisk

## Discussion

This study showed that patients exhibiting patellar maltracking after the implantation of TKA components had higher lateral PFP in the flexed knee position than those exhibiting favorable patellar tracking. Additionally, this study revealed that LRR for patients with patellar maltracking reduced the lateral PFP at knee flexion and consequently improved patellar tracking, as hypothesized.

Previous studies showed that measuring the PFP was useful for the objective evaluation of patellar tracking in TKA. Hsu et al. investigated the PFP during knee range of motion (ROM) using a uniaxial force transducer embedded inside a patellar component in cadaveric specimens; they reported that the PFP after TKA was consistently higher than that of the native knee and that LRR after TKA decreased the PFP at a greater knee flexion angle [32]. Konno et al. evaluated the relationship between the tibiofemoral kinematic pattern after navigation-assisted TKA and the PFP measured by an ultrathin force transducer in a clinical situation; they reported that the medial pivot pattern resulted in a reduced PFP and improved patellar tracking [33]. These studies calculated the PFP as the total force on the sensor but did not evaluate the distribution of the PFP. Several studies have evaluated retinacular tension and patellar tracking by measuring the mediolateral balance of the PFP. Peretz et al. [9] and King et al. [10] measured the maximal PFP during complete knee ROM of half-body cadavers using a pressure sensor sewn to the patellar articular surface. They demonstrated that the implantation of standard TKA components increased the maximal lateral PFP and decreased the maximal medial PFP compared with the native knee. However, subsequent LRR after TKA decreased the differentials between the lateral and medial counterpart. They confirmed that patellar maltracking was attributed to the increased lateral PFP compared with the medial counterpart and that LRR corrected the imbalance of the PFP and consequently improved patellar tracking. The results of the present study support the findings of those previous studies in clinical settings.

The current study demonstrated that the lateral PFP in the flexed-knee position was significantly correlated with the PRA and ISR in both the RP and RPK phases. The results of this study indicated that internal rotational patellar resection, which led to a thick medial patellar remnant and a thin lateral counterpart (Fig. 5D), and patella alta were causative factors for high lateral PFP, which induced patellar maltracking after TKA.

Several studies have investigated the relationship between patellar tracking and the PRA; nonetheless, the effect of the PRA on patellar tracking remains controversial. White et al. measured the position of patellar component using postoperative radiographs obtained from 90 patients who underwent TKA and investigated its effect on the clinical outcomes of patellofemoral-related complications [26]. They reported that a PRA of more than 5° of internal rotation (PRA < −5°) was a risk factor for the development of AKP and painless noise. In our study, 3 out of 44 cases were found to have a PRA of < −5°, and all of these three cases exhibited patellar maltracking after the implantation of TKA components, which supports the findings of White et al. A recent study by Kleeman et al. investigated the bone strain at the patellar remnant for different PRAs, ranging from −7° to 7°, in their computational analysis [34]. They demonstrated that increasing the internal rotation of the PRA resulted in an increase in the strain energy density of the patellar bone. Furthermore, the highest strain energy density was observed with the thinnest lateral facet remnant. These results suggested that the internal rotational patellar resection increased PFP, although its mediolateral balance was not clarified. Meanwhile, the results of our study showed that the PRA significantly correlated with not only the lateral PFP in the RPK phase at 120° (*rs* = −0.47, *p* = 0.0015) and 135° (*rs* = −0.40, *p* = 0.0078) but also the sum of the lateral and medial PFP in the RPK phase at 120° (*rs* = −0.48, *p* = 0.0010) and 135° (*rs* = −0.31, *p* = 0.045) of knee flexion. In other words, we demonstrated that internal rotational patellar resection increased the total PFP as well as the lateral PFP after TKA; these findings support those of Kleeman et al. In contrast, a few studies reported that internal rotational patellar resection had a beneficial effect on patellar tracking [31, 35]. Kawano et al. measured the PT on postoperative radiographs taken from 62 patients who underwent CR-type TKA as a parameter of patellar tracking and showed that the PRA was significantly correlated with the PT [31]. They confirmed that internal rotational patellar resection reduced the lateral PT, which was advantageous for gaining optimal patellar tracking. However, these results should be interpreted with caution because their assessment of patellar tracking was not performed intraoperatively but was instead conducted postoperatively on the basis of radiographic parameters. Forty out of 62 patients in their study underwent LRR, which might have substantially changed patellar tracking and consequently altered the PT on postoperative radiographs. Therefore, their study might not have thoroughly verified the effect of the PRA on patellar tracking. Another reason for the controversy regarding the effect of the PRA on patellar tracking is that there exists no consensus on how the neutral patellar resection plane should be determined. While several methods for determining the neutral patellar resection plane on the skyline view have been reported, the reproducibility of measurement significantly differs among these methods, thereby causing difference in the reproducibility of PRA determination [30, 35]. Furthermore, some previous studies did not describe or define the neutral resection plane [29, 31]. Therefore, heterogeneity of the effect of the PRA on patellar tracking might have been attributable to the discrepancy in the method for determining the neutral patellar resection plane among previous studies. In contrast, we adopted a medial-divot method [30] to define the neutral patellar resection plane and achieved high reproducibility of PRA measurement, which is one of the strengths of our study.

Patellar height has been reported to be related to the PFP. Luyckx et al. measured the PFP in PS-type TKA at the setting of different patellar heights with a dynamic knee simulator and showed that patella alta was associated with higher PFP than that of normal patellar height at deeper knee flexion (70–120°) [11]. Furthermore, Innocenti et al. reported that the same tendency of the effect of the patellar height on the PFP was consistently observed for different TKA types even though each TKA type had different magnitudes of PFP [36]. In addition, a recent computational study conducted by Tischer et al. also shown that patella alta resulted in a 16% higher PFP, as compared with the normal patellar height, after CR-type TKA with resurfaced patella [12]. Our results are consistent with the findings of these previous studies. However, there was only a weak correlation between ISR and the lateral PFP at no less than 90° knee flexion, whereas PRA showed a moderate correlation with the lateral PFP at no less than 120° knee flexion. Therefore, patella alta may have a relatively small effect on increased lateral PFP.

The current study had some limitations. First, we used both CR-type (33 cases) and MC-type (11 cases) bearings, and this may have affected the PFP. A previous cadaveric study assessed the influence of TKA-type on the patellar biomechanics and the PFP [37]. They demonstrated that the PFP was largest in condylar-stabilizing TKA, followed by in order CR- and PS-type TKA. Meanwhile, we compared the lateral PFP at 0°, 90°, 120°, and 135° knee flexion between CR- and MC-type bearings using the Mann–Whitney *U* test. The result showed that there was no significant difference in the lateral PFP between CR and MC-type bearings at 0° (2.4 ± 1.9 N versus 3.4 ± 2.3 N, *p* = 0.16), 90° (34.5 ± 23.2 N versus 47.8 ± 21.6 N, *p* = 0.13), 120° (47.8 ± 26.2 N versus 63.6 ± 20.0 N, *p* = 0.065) and 135° (57.6 ± 31.0 N versus 70.4 ± 24.2 N, *p* = 0.33). However, post-hoc power analysis showed that our sample size did not achieve a power of ≥ 80% with a two-sided alpha of 0.05 (effect size 0.674, power 45.4%). Therefore, our small sample size may have increased the likelihood of type II errors (i.e., the inability to detect the true effect of the difference in bearing type on the lateral PFP). As a result, future studies with larger sample sizes are required. Second, the strength of the quadriceps muscle was not evaluated. As indicated by previous reports, the lower strength of the vastus medialis relative to the vastus lateralis shifts the center of the PFP laterally on the patella and induced abnormal patellar tracking [38, 39]. No significant differences in demographic characteristics, such as age, sex, body mass index, and OA severity, were observed between the FT and IT groups in this study; nevertheless, the difference in the preoperative strength of the quadriceps muscles among patients might have had some effect on the PFP and the patellar tracking. Third, the diameter of the custom-made patellar component with which we measured the PFP was 32 mm, which was larger by 3 mm than that of patellar components actually implanted in 27 cases. These differences might have altered the PF kinematics and consequently changed patellar tracking and PFP. Fourth, we did not conduct the radiographic measurement of the tibial and femoral component rotation using postoperative CT images or radiographs. Malrotation of the TKA component might have had some negative effect on the PFP and the patellar tracking if the TKA components had not been accurately inserted with the relevant rotation, as planned. Fifth, there is a possibility that the slight deterioration of the fixation property of the patellar component might progress with the repetition of the PFP measurement, thereby affecting the accuracy of the PFP measurement. Sixth, the effect of LRR performed distally to the mid-patellar level on the PFP was not investigated. We selectively extended LRR according to previous studies [13] for the six patients who demonstrated impaired patellar tacking despite primary LRR, in which the lateral retinacular tissues from the mid-level of the patella distally to the joint line were resected. Although all of the six cases achieved favorable patellar tracking following extended LRR, we did not evaluate the PFP in the phase when the procedure was performed. Further studies should be conducted to verify the effect of stepwise LRR on the PFP. Lastly, it is unclear whether high lateral PFP negatively affects postoperative clinical outcomes. A previous study investigated the influence of high PFP on postoperative clinical outcomes and reported that the high PFP at 60° and 140° knee flexion was associated with the deterioration of patient satisfaction, Patella score and Forgotten Joint Score-12 at 2 years postoperatively [40]*.* The results of our study suggest that cutting the patella in the externally rotated plane reduces the lateral PFP and, consequently, is advantageous for gaining optimal patellar tracking in patellar-resurfacing TKA. However, we did not investigate the effects of this type of asymmetric patellar resection and decrease of the lateral PFP on clinical outcomes. Furthermore, asymmetric patellar resection may increase the risk of postoperative complications, such as patellar fracture, component loosening, and component wear [41–43]; thus, its effect on postoperative outcomes is conflicting. Further follow-up studies investigating the relationship among the PFP, PRA, and clinical outcomes, including postoperative complications, are required.

## Conclusions

The current study demonstrated that internal rotational patellar resection, which resulted in a thick medial patellar remnant and a thin lateral counterpart, and patella alta were the causative factors of high lateral PFP in the knee flexion position, which induced patellar maltracking after TKA.

Therefore, surgeons should avoid internal rotational patellar resection to achieve favorable patellar tracking and perform LRR in patients with patellar maltracking.

## Data Availability

The datasets generated and/or analyzed during the current study are available from the corresponding author on a reasonable requests.

## Abbreviations

AFO: Anterior femoral offset
AKP: Anterior knee pain
CR: Cruciate-retaining
CT: Computed tomography
FCRA: Femoral component rotational angle
FT: Favorable patellar tracking
ICC: Intraclass correlation coefficient
ISR: Insall–Salvati ratio
IT: Impaired patellar tracking
KL: Kellgren–Lawrence
LRR: Lateral retinacular release
MC: Medial-congruent
OA: Osteoarthritis
PCA: Posterior condylar angle
PCL: Posterior cruciate ligament
PFP: Patellofemoral pressure
PRA: Patellar resection angle
PS: Posterior-stabilized
PT: Patellar tilt
ROM: Range of motion
RP: Resurfaced patella
RPK: Resurfaced patella and knee
TKA: Total knee arthroplasty
